# Differential Regulation of Inflammation and Immunity in Mild and Severe Experimental Asthma

**DOI:** 10.1155/2013/808470

**Published:** 2013-05-27

**Authors:** Seil Sagar, Kim A. T. Verheijden, Niki A. Georgiou, Johan Garssen, Aletta D. Kraneveld, Arjan P. Vos, Gert Folkerts

**Affiliations:** ^1^Division of Pharmacology, Utrecht Institute for Pharmaceutical Sciences, Faculty of Science, Utrecht University, P.O. Box 80082, 3508 TB Utrecht, The Netherlands; ^2^Danone Research, Centre for Specialised Nutrition, 6704 PH Wageningen, The Netherlands

## Abstract

This study aimed at exploring innate and adaptive immunity in allergic asthma by investigation of mRNA expression of pattern recognition receptors, T-cell-specific cytokines, and transcription factors. Mouse models for mild and severe asthma, with similar pathological characteristics observed in humans, were used to study the involved inflammatory markers as a first step in the development of phenotype-directed treatment approaches. In the mild model, mice were sensitized to ovalbumin-Imject Alum and challenged with ovalbumin. In the severe model, mice were sensitized to trinitrophenyl-conjugated ovalbumin and challenged with trinitrophenyl-ovalbumin/IgE immune complex. Pulmonary airway inflammation and mRNA expression of Toll-like receptors (TLRs), NOD-like receptors (NLRs), T cell cytokines, and transcription factors in lung tissue were examined. Different mRNA expression profiles of TLRs, NLRs, T cell cytokines, and transcription factors were observed. In the mild model, *Il10* showed the largest increase in expression, whereas in the severe model, it was *Inf* **γ** with the largest increase. Expression of *Tbet* was also significantly increased in the severe model. Inflammation and immunity are differentially regulated in mild and severe experimental asthma. This preclinical data may help in directing clinical research towards a better understanding and therapy in mild and severe asthmatic patients.

## 1. Introduction

Asthma is a chronic inflammatory disease of the airways affecting over 300 million people worldwide, and its prevalence is rising, especially in children and within developing countries [1]. New studies indicated that in allergic disorders, including asthma, innate immunity is deregulated by allergens that promote sensitization [2]. However, the underlying immunological processes are still not fully understood as asthma is a complex multifactorial disease in which both innate and adaptive immune responses are involved [3]. In addition, asthma is considered as a complex syndrome with different clinical phenotypes in children and adults. Eosinophils and neutrophils play a key role in the cellular airway inflammation [4, 5]. The different phenotypes of asthma have distinct immunological and pathological features. Mild asthma is characterized by chronic inflammation of the airways that is mostly eosinophilic in nature and allergic sensitization. The resulting airway inflammation is thought to be caused by a breakdown of immune tolerance toward environmental antigens and leads to a T helper type 2(Th2)-biased immune response [6]. On the contrary, neutrophil accumulation in the bronchial mucosa is an important feature of severe asthma and frequently includes a T helper type 1(Th1) component as well as a Th2 immune response [4, 6]. This heterogeneity highlights the importance of more specific treatment approaches based on asthma phenotypes. 

Pattern recognition receptors (PRRs), like the Toll-like (TLRs) and NOD-like (NLRs) families of receptors, are key components of the innate immune system. These PRRs exhibit different cell and stimulus-specific patterns of expression [7]. In the human airways, TLRs are expressed in and on dendritic cells (DCs), epithelial cells, eosinophils, macrophages, and mast cells [8]. NOD1 and NOD2 are intracellular pattern recognition molecules (PRMs) expressed in various human epithelial cells including lung cells [9]. Multiple DC functions are controlled by PRRs and, ultimately, modulate the resulting adaptive immune response [8, 10]. Upon PRR activation in the lung, various chemokines and cytokines are produced by mast cells and eosinophils that recruit activated B-lymphocytes and Th lymphocytes to the lung, starting the inflammation process in the airways [11]. 

Mast cells express the high-affinity receptors (Fc*ε*RI) for immunoglobulin E (IgE) on their surface, and animal studies have demonstrated that mast cells play an important role in the induction of allergic airway inflammation [12]. In addition, asthmatic patients have been shown to have an increased number of lung mast cells and allergen-specific IgE, a phenomenon of allergic asthma shared with mouse models for this disease [13]. In order to reproduce more of the clinical features of severe asthmatic patients, there has been a focus on using IgE immune complexes as inducers of immune responses in the murine lung. Trinitrophenyl-(TNP-) ovalbumin-(OVA-) IgE immune complexes have been shown to be more potent inducers of immune responses than antigens alone, since challenge of sensitized mice with these complexes resulted in an increased migration of mast cell progenitors to the lung [12, 13]. 

The expression and function of PRRs have been linked to susceptibility towards allergic asthma [2, 14]. Functional genetic variations in *TLR1*, *TLR6*, and *TLR10 *genes affecting gene and protein expression have been shown to be associated with increased mRNA expression of these TLRs and to protect against atopic asthma in humans [14]. Single nucleotide polymorphisms (SNPs) in the *TLR2* gene that led to decreased mRNA expression were positively associated with asthma susceptibility [15]. Cord blood CD34 (+) cells from high-atopic-risk infants exhibited low *TLR2, TLR4*,* and TLR9 *expressions [16]. Additionally, amino acid changes in the *TLR2* gene have been linked to reduced *TLR2 *receptor function and to increase in atopy risk in humans [17]. *Tlr3* contributes to asthma exacerbation in mice [18]. A study in a murine macrophage cell line suggested a proinflammatory role of *Tlr4 and 5* in the disease [19]. Animal studies have demonstrated that the dose of the *Tlr4 *ligand, lipopolysaccharide (LPS), determines the type of inflammatory response generated and that lung epithelial cells activation by *Tlr4* is crucial for induction of airway inflammation via activation of mucosal DCs [20–22]. *TLR7* and *8* were identified as novel risk genes for asthma [23]. *TLR9* is one of the most extensively studied TLRs in asthma, and it is currently thought to modulate allergic responses by skewing the balance from a Th2 towards a Th1 response [24]. In addition, SNPs in the *TLR9* gene were associated with increased risk of asthma [25]. *TLR11*, *12*, and *13* are not encoded in the human genome, and there are currently no data on associations with asthma in mice [14]. Insertion-deletion polymorphisms in the *NOD1* gene have been associated with increased risk of developing asthma, and genetic variations in *NOD1 *that affected microbial recognition were positively associated with disease susceptibility and pathogenesis [26, 27]. Polymorphisms in *NOD2,* that affected LPS recognition and *TLR4* function, were associated with atopic diseases and were suggested to indirectly increase the severity of asthma [28]. 

In asthma, over 50 cytokines have now been identified to determine disease outcome. Proinflammatory and Th2-associated cytokines, including interleukin-4 (IL-4), IL-5, IL-6, IL-13, and tumor necrosis factor *α* (TNF-*α*), are reported to enhance the disease. On the other hand, interferon-*γ* (IFN*γ*), a Th1-associated cytokine, was reported to reduce the symptoms of asthma in asthmatic patients [11]. In addition, asthmatic patients have been shown to have reduced levels of the anti-inflammatory cytokine IL-10 in the sputum. IL-10 is produced by macrophages and by a subset of regulatory T cells (Tregs) and exerts its effects by inhibiting the synthesis of inflammatory cytokines (including asthma-associated cytokines such as TNF-*α* and IL-5) and gene presentation [29]. Th2 cells play a key role in asthma, and asthmatic subjects have been reported to have Th1/Th2 imbalances as well as disturbed T helper type 17 (Th17)/Treg balances [30]. Each Th cell type is regulated by a specific transcription factor: Tbet for Th1 cells, GATA-3 for Th2 cells, retinoic acid orphan receptor-*γ*t (ROR*γ*t) for Th17 cells, and forkhead box P3 (Foxp3) for Tregs [31]. Animal and human studies have demonstrated that alterations in expression and/or of functions of these transcription factors can contribute to asthma pathogenesis [32, 33].

The aim of this current study is to explore the innate and adaptive immune responses and inflammation in allergic asthma by investigation of the mRNA expression profiles of the different PRRs, T cell-related cytokines, and transcription factors. To this end, we have used a mouse model for both mild allergy and severe asthma with similar pathological characteristics seen in humans. Findings from this observational study may contribute to elucidating the underlying mechanisms of mild and severe asthma and the involved inflammatory markers, as a first step in the development of phenotype-directed treatment approaches. 

## 2. Material and Methods

### 2.1. Animals

Male BALB/c mice (6–8 weeks; Charles River Laboratories, France) were acclimated to their new environment for at least 1 week before the start of the experiment. Mice were housed under standard conditions and had free access to food and water. All *in vivo *experiments were approved by and were in accordance with the guidelines of the local Dutch Committee of Animal Experimentation.

### 2.2. Mild Asthma Model

#### 2.2.1. OVA Sensitization

**Figure 1:**
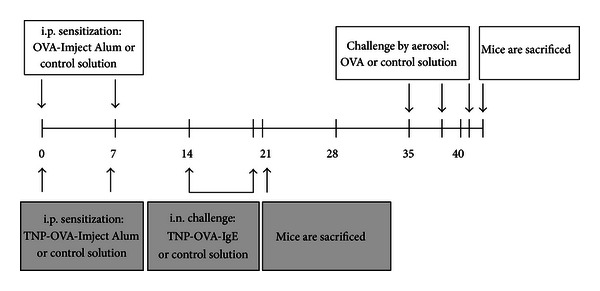
Time schedule of the mild and severe allergic asthma mouse models. Mild model (white): male BALB/c mice were sensitized intraperitoneally with alum-precipitated OVA or saline only on days 0 and 7 and challenged with OVA or saline aerosol on days 35, 38, and 41; mice were sacrificed on day 42. Severe model (grey): male BALB/c mice were sensitized intraperitoneally with alum-precipitated TNP-OVA or saline only on days 0 and 7 and challenged intranasally with TNP-OVA/IgE immune complex or saline from day 14 up to and including day 20; mice were sacrificed on day 21.

Sensitizations were performed on days 0 and 7. Mice were sensitized to ovalbumin (OVA; chicken egg albumin, grade V, Sigma, St. Louis, MO, USA) by intraperitoneal injections of 0.1 mL alum-precipitated antigen, comprising 10 *μ*g OVA absorbed into 2.25 mg alum (Imject Alum; Pierce, Rockford, IL, USA). Control animals received 0.1 mL saline only (NaCl 0.9%; B. Braun Medical BV, Oss, The Netherlands) (Figure 1). 

#### 2.2.2. OVA Challenge

Mice were exposed to 10 mg/mL OVA aerosol in saline using Pari LC Star nebulizer (PARI GmbH, Starnberg, Germany) in an aerosol cabin for 30 min on days 35, 38, and 41. Control animals were exposed to nebulized saline aerosol only (Figure 1). 

### 2.3. Severe Asthma Model

#### 2.3.1. TNP-OVA Sensitization

Sensitizations were performed on days 0 and 7. Mice were sensitized with trinitrophenyl-(TNP-) conjugated OVA by intraperitoneal injections of 0.1 mL alum-precipitated antigen, comprising 10 *μ*g TNP-OVA absorbed into 2.25 mg alum. Control animals received 0.1 mL saline only (Figure 1). 

#### 2.3.2. TNP-OVA-IgE Challenge

From day 14 up to and including day 20, mice were challenged daily by intranasal administration of a TNP-ovalbumin/IgE immune complex (2 *μ*g TNP-OVA plus 20 *μ*g DNP-specific IgE (clone H1 26.82)), as described previously [34]. Control animals received 50 *μ*L of saline only (Figure 1). 

### 2.4. Bronchoalveolar Lavage

After sacrifice, on day 42 (mild model) or 21 (severe model), lungs were first washed through a tracheal cannula with 1 mL saline containing protease inhibitor cocktail (Complete Mini, Roche Diagnostics, Mannheim, Germany) and prewarmed at 37°C. This was repeated 3 times with 1 mL saline only. Cytospin cell preparations were made by cytospinning the cells onto glass for 5 min (400 g, 4°C), and cytospins were stained by Diff-Quick (Merz and Dade AG, Düdingen, Switzerland). Numbers of eosinophils, macrophages, neutrophils, and lymphocytes were scored by light microscopy. 

### 2.5. RNA Isolation and Quantitative Real-Time PCR

After mice were sacrificed, on day 42 or 21, the lungs were dissected, and mRNA was isolated from whole lung tissue. Messenger RNA isolation (*n* = 3 mice per group) was carried out according to the Qiagen RNeasy Mini Kit protocol (Qiagen Benelux BV, Venlo, the Netherlands). Reverse transcriptase PCR was performed using an iScript cDNA Synthesis Kit (Bio-Rad Laboratories, Hercules, CA, USA). The reactions were performed in a PTC-100TM Programmable Thermal Controller (M. J. Research Inc., Waltham, MA, USA) according to manufacturer's protocol.

cDNA was amplified using iQ SYBR Green Supermix in a 96-well PCR plate and run in a CFX96 Real-Time PCR Detection System (Bio-Rad). Primers for TLRs, NLRs, ribosomal protein S13 (RPS13, reference gene), and T-cell transcription factors were purchased by Isogen (Isogen Life Science, De Meern, The Netherlands). The Sequences are listed in Supplementary Table 1 available online at http://dx.doi.org/10.1155/2013/808470. For mouse T-cell cytokines, RT² qPCR Primer Assays (SABiosciences, Venlo, The Netherlands) were used. The protocol used for amplification was 94°C for 3 min, 94°C for 10 sec, and specific melt temperature for 45 sec, followed by 39 cycles of 94°C for 10 sec and 95°C for 10 sec.

Normalized gene expression (ΔΔC_T_) was calculated using the built-in gene expression analysis module in CFX Manager software (CFX Manager software version 1.6). 

### 2.6. Statistical Analysis

Data analysis was performed using a 1-way analysis of variance (one-way ANOVA) with the Bonferroni's post hoc test. In some studies, the Student's *t*-test was used. Linear regression analysis was used to calculate correlations. All statistical analyses were performed using the GraphPad Prism software program (GraphPad Prism software version 5.03). 

## 3. Results

### 3.1. Allergen-Sensitized and Challenged Mice in the Severe Asthma Model Show Higher Total Inflammatory Cell Number

**Figure 2:**
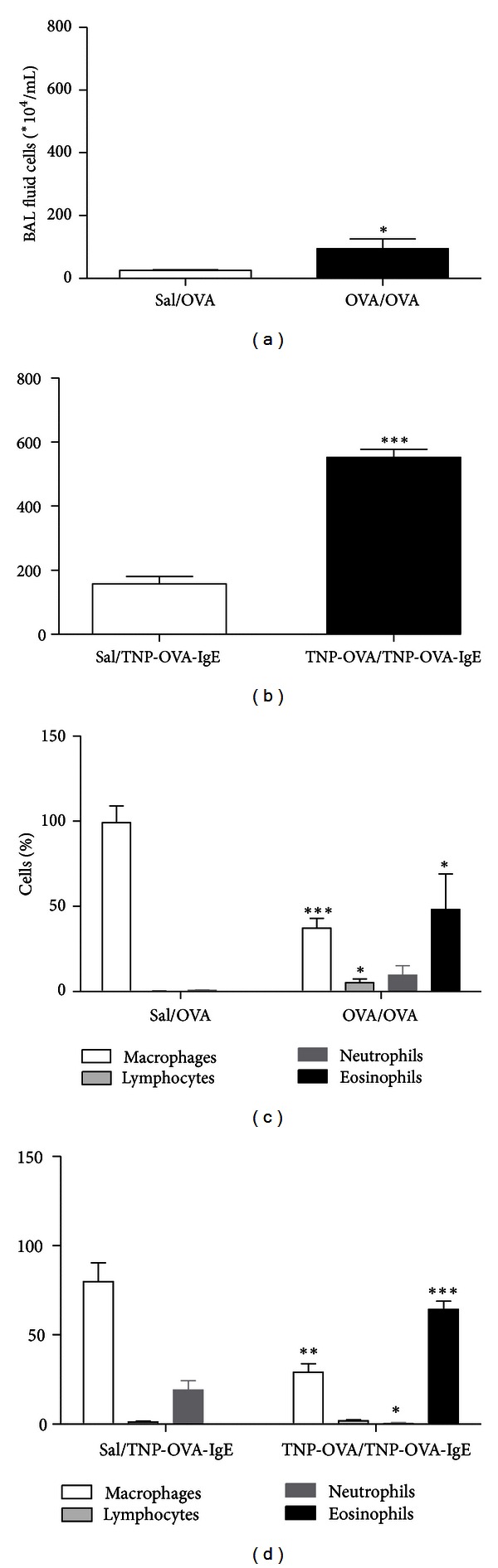
Total and differential BAL fluid cell counts in mild and severe allergic asthma. The total inflammatory cell number in BAL fluid is shown in (a) mild model and (b) severe model. The differential cell counts are shown as percentages of the total cell count in (c) mild model and (d) severe model. Results are presented as mean ± SEM, *n* = 9 mice/group. Student's *t*-test was used to compare the means of the OVA-sensitized and challenged mice with the means of only challenged mice for each cell type, *P < 0.05; **P < 0.01; ***P < 0.0001.

Mice were rendered asthmatic following the scheme presented in Figure 1. To examine the extent of pulmonary inflammation in the asthmatic mice, bronchoalveolar lavage (BAL) fluid was examined for leukocyte accumulation (Figure 2). In mild asthma, allergen-sensitized and challenged mice showed a significant increase in the total inflammatory cell number (Figure 2(a)) which was due to a relative increase in the number of lymphocytes and eosinophils (Figure 2(c)) in BAL fluid compared to challenged only mice. In severe asthma, a significantly higher total inflammatory cell number (Figure 2(b)) was observed in allergen-sensitized and challenged mice compared to challenged only mice, and this was due to a relative increase in the number of eosinophils (Figure 2(d)). 

### 3.2. TLR and NLR mRNA Expression in Lung Tissue Is Differentially Modulated in Mild and Severe Asthma Models

**Figure 3:**
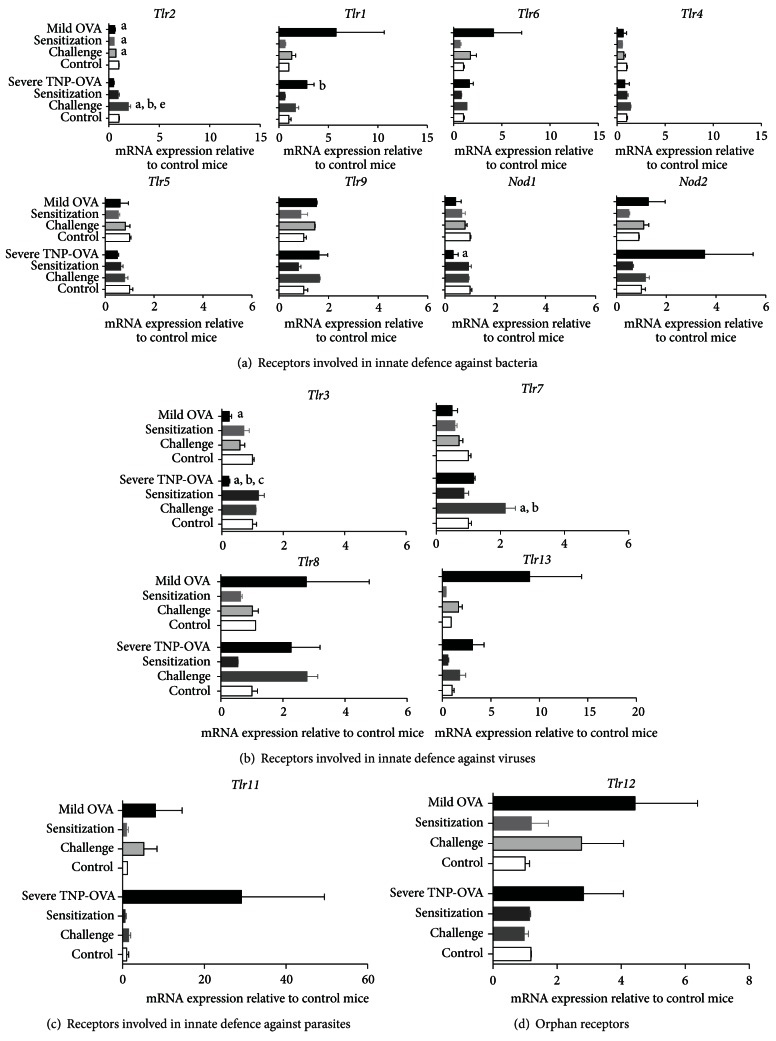
Relative TLR and NLR mRNA expression in mouse whole lung tissue during mild and severe allergic asthma. The results are presented as mRNA expression levels relative to control mice. Data is shown as mean ± SEM. Statistical significance of differences was tested using Bonferroni's post hoc test after one-way ANOVA. ^a^ *P* < 0.05 compared to control mice; ^b^ *P* < 0.05 compared to sensitized only mice; ^c^ *P* < 0.05 compared to challenged only mice; and ^e^ *P* < 0.05 compared to OVA-sensitized and challenged mice.

As PRRs in the lung can modulate ongoing chronic inflammation during asthma, the mRNA expression of *Tlr1-13* and *Nod1 and 2* was measured (Figure 3). In the mild model, *Tlr2* expression was significantly increased in control mice when compared to sensitized only, challenged only, and sensitized and challenged mice (Figure 3(a)). In the severe model, *Tlr2 *expression was significantly increased in challenged only mice when compared to control, sensitized only, and allergen-sensitized and challenged mice (Figure 3(a)). In addition, sensitized and challenged mice showed significantly higher *Tlr1* expression when compared to sensitized only mice and significantly lower *Nod1 *expression in comparison to control mice (Figure 3(a)). In mild asthma, *Tlr3 *expression was significantly decreased in allergen-sensitized and challenged mice when compared to control mice (Figure 3(b)). In severe asthma, *Tlr3* expression was significantly lower in sensitized and challenged mice but not in control, sensitized only, and challenged only mice (Figure 3(b)). In addition, *Tlr7* expression was significantly higher in challenged only mice when compared to control and sensitized only mice (Figure 3(b)). The expression of *Tlr11 *and *Tlr12 *remained unchanged in both models (Figures 3(c) and 3(d)). Interestingly, in the severe model, the mRNA expression of *Tlr1*, *Tlr3*, *Tlr6*, *Tlr9*, *Tlr11*, *Tlr13*, *Nod1*, and *Nod2* was significantly correlated with the total inflammatory cell number in BAL fluid (Table 1). 

### 3.3. Allergen Sensitization and Challenge in the Severe Asthma Model Enhance the mRNA Expression of *Il5*, *Il6*, and *Il10* in Lung Tissue

To determine the extent of inflammation in the lung, the mRNA expression of various cytokines was measured (Table 2). In the severe model, the expression of almost all cytokines tended to be increased in TNP-OVA-sensitized and TNP-OVA/IgE-challenged mice when compared to OVA-sensitized and challenged mice in the mild model. *Il10* expression was significantly increased in both models. In severe asthma, an 8 and 33-times higher *Il5* and *Il6* expressions, respectively, was observed in allergen-sensitized and challenged mice but not in control, sensitized only, and challenged only mice. 

### 3.4. *Cytokine mRNA* Expression in Lung Tissue Is Differentially Modulated in Mild and Severe Asthma

**Figure 4:**
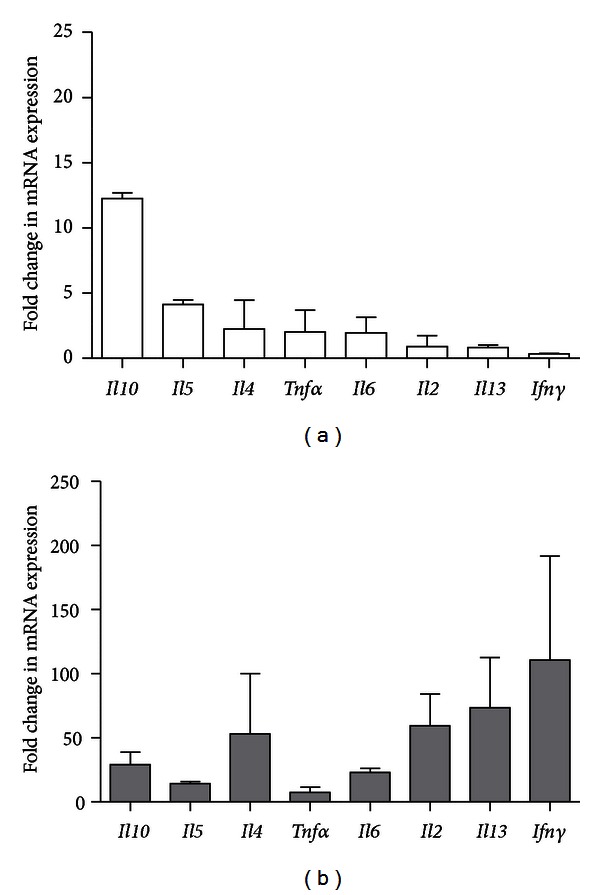
Change in T-cell *cytokine mRNA* expression in mouse whole lung tissue during mild and severe allergic asthma: (a) mild model and (b) severe model. Data is shown as mean ± SEM. Fold change is calculated by dividing the *cytokine mRNA* expression in OVA-sensitized and challenged mice by *cytokine mRNA* expression in challenged only mice.

In mild asthma, *Il10* showed the largest change in expression followed by *II5, II4*, *Tnf*α**, *Il6*, *Il2*, *Il13*, and *Ifn*γ*,* respectively, (Figure 4(a)). In severe asthma, the largest change (110-fold) is observed in *Ifn*γ** expression followed by *Il13*, *Il2*, *Il4*, *Il10*, *Il6*,* Il5*, and *Tnf*α*,* respectively (Figure 4(b)). 

### 3.5. Allergen Sensitization and Challenge in the Severe Asthma Model Result in a Strong Upregulation of mRNA for *Tbet* and *Foxp3* in the Lungs and Skews the Immune Response Away from Th2 and towards Treg

To examine the T cell responses in the lung, the mRNA expression of T cell-specific transcription factors was measured, and to determine the extent of Th response skewing in the lung, ratios for *Gata3/Tbet *(Th2/Th1),* Foxp3/Ror*γ*t* (Treg/Th17)*, Foxp3/Gata3 *(Treg/Th2), and* Foxp3/Tbet *(Treg/Th1) mRNA expressions were calculated (Table 3). The expression of *Tbet* and *Foxp3* was 7 and 2-times higher, respectively, in allergen-sensitized and challenged mice in the severe model when compared to allergen-sensitized and challenged mice in the mild model. In severe asthma, sensitized and challenged mice showed 16 and 17-times higher* Tbet* and *Foxp3* expression, respectively, when compared to control, sensitized only and challenged only mice. Most interestingly, allergen sensitization and challenge resulted in a significant decrease in *Gata3*/*Tbet *ratio as compared to control and sensitized only mice in both models. The ratio of *Foxp3/Ror*γ*t *was 12-times higher in the severe model in comparison to the mild model, and this ratio was significantly increased in allergen-sensitized and challenged mice when compared to control, sensitized only, and challenged only mice in both models. In the mild model, allergen sensitization and challenge significantly increased the *Foxp3/Gata3 *ratio when compared to control mice. The ratio of *Foxp3/Gata3 *in allergen-sensitized and challenged mice was significantly increased when compared to control, sensitized only, and challenged only mice, and this ratio was almost 4-times higher than the mild model.

### 3.6. T Cell Transcription Factors mRNA Expression in Lung Tissue Is Differentially Modulated in Mild and Severe Asthma

**Figure 5:**
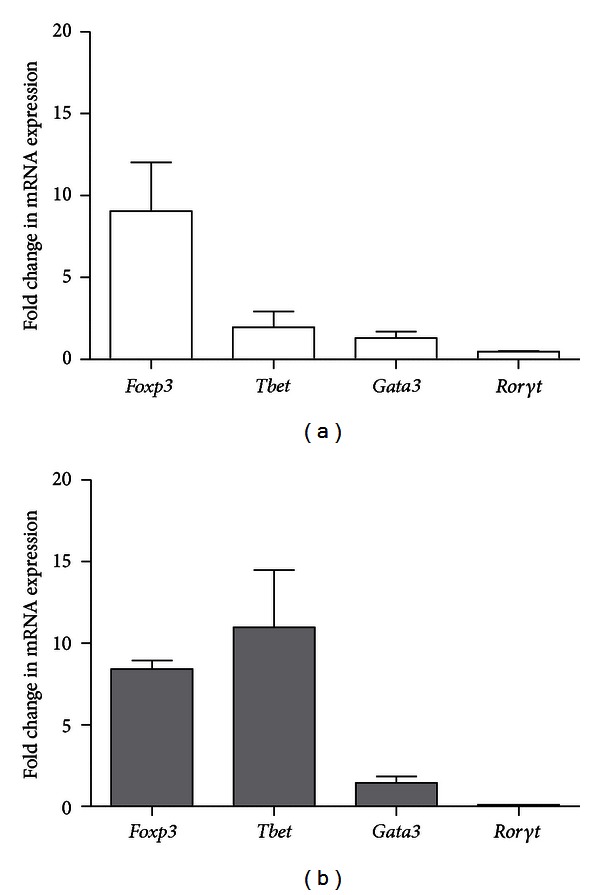
Change in T-cell transcription factor mRNA expression in mouse whole lung tissue during mild and severe allergic asthma: (a) mild model and (b) severe model. Data is shown as mean ± SEM. Fold change is calculated by dividing the transcription factor mRNA expression in OVA-sensitized and challenged mice by *cytokine mRNA* expression in challenged only mice.

In mild asthma, the largest change (9-fold) was observed in *Foxp3* expression followed by *Tbet*, *Gata3*, and *Ror*γ*t *(Figure 5(a)). *Tbet* showed the largest change in expression in severe asthma followed by *Foxp3*, *Gata3*, and *Ror*γ*t *(Figure 5(b)). 

### 3.7. Different Correlations between T-Cell Transcription Factors mRNA Expression and T-Cell *Cytokines mRNA* Expression Are Found in Mild and Severe Asthma

As imbalances in T cell responses can also be detected using T cell-specific transcription factors, the correlations between the mRNA expression of T cell transcription factors and T cell-specific cytokines were calculated (Table 4). In mild asthma, *Tbet* expression was significantly correlated with *Il2*, *Il4*, *Il5*, *Il6*, *Il13,* and *Tnf*α** expression. *Ror*γ*t* expression showed a significant correlation with *Il10* expression, and *Foxp3* expression was significantly correlated with and* Il2*, *Il4*, *Il5*, *Il6*, *Il10,* and *Tnf*α** expression. In severe asthma, *Tbet* expression was strongly correlated with *Il2*, *Il6,* and *Il10* expression. *Ror*γ*t* expression was significantly correlated with *Il2*, *Il4*, *Il5*, *Il6*, *Il10,* and *Il13* expression. Interestingly, *Foxp3* expression was strongly correlated with *Il2*, *Il4*, *Il5*, *Il6,* and *Il13* and significantly correlated with *Il10* and *Ifn*γ** expression. 

## 4. Discussion 

The aim of this study was to explore the innate and adaptive immune responses and inflammation in mouse models for mild and severe allergic asthma. Our results clearly show that pulmonary inflammation is differentially regulated in mild and severe experimental asthma. In the severe asthma model, a higher (3-fold) cell influx in BAL fluid was seen compared to the mild model. As expected, TNP-OVA-sensitized and TNP-OVA/IgE-challenged mice showed significantly higher total inflammatory cell number and a relative increase in the number of eosinophils, lymphocytes, and neutrophils as compared to challenged only mice. These findings are in accordance with other animal studies in which IgE immune complexes have been used as inducers of airway inflammation [12, 13, 34]. 

Besides being key components of the innate immunity, PRRs are also involved in the activation and shaping of adaptive immunity. The function and expression of PRRs have been linked to susceptibility towards allergic asthma. In mild asthma, *Tlr2 *expression was lower in sensitized only, challenged only and OVA-sensitized and challenged mice, when compared to the control mice. Previous human studies have demonstrated that decreased *TLR2 *mRNA expression and receptor function due to SNPs in the *TLR2 *gene are positively associated with asthma susceptibility, and high-atopic-risk infants have been reported to have low *TLR2* expression on their cord blood CD34 (+) cells [15–17]. In severe asthma, allergen challenge only increased the expression of *Tlr2* and allergen sensitization and challenge significantly increased *Tlr1* expression when compared to sensitized only mice. These results are supported by data from animal and human studies which have shown that *Tlr2/Tlr1* heterodimers can play both pro- and anti-inflammatory roles in allergic asthma [16, 35]. Interestingly, allergen sensitization and challenge decreased *Tlr3 *expression in both models. Previous *in vitro* studies have demonstrated that upon the activation of *Tlr3 *by its ligand, this PRR induces upregulation of its own expression as well as expression of other TLRs and various cytokines and chemokines and thereby contributes to exacerbation of inflammation [18]. However, no direct associations between *Tlr3* expression and function and asthma have been reported yet, and whether the decreased mRNA expression of *Tlr3* caused by the chronic inflammatory status of the animals is proinflammatory or anti-inflammatory is also unknown. In severe asthma, allergen challenge increased *Tlr7* expression. This could be due to a response of plasmacytoid DCs to the inhaled antigen as these cells strongly express *Tlr7* [36]. In addition, allergen sensitization and challenge decreased *Nod1* expression when compared to control mice. *Nod1* is an intracellular sensor of pathogenic bacteria. Single nucleotide polymorphisms in the *Nod1* gene were positively associated with susceptibility towards asthma in children living on farms, and this PRR has been reported to be necessary for neutrophil function in mice [26, 27, 37]. However, no direct associations between *Nod1 *expression and function and asthma have been reported yet. 

Interestingly, *Tlr1*, *Tlr3*, *Tlr6*, *Tlr9*, *Tlr11*, *Tlr13*, *Nod1*, and *Nod2* expression was significantly correlated with the total inflammatory cell number in BAL fluid in the severe model. These correlations might be explained by the increase in the number of inflammatory cells which express these receptors, as is the case for *Tlr1* and *Nod1*. Additionally, these receptors could also contribute to the increased sensitivity to inflammation/exacerbation since a small trigger can lead to an inflammatory cascade. However, the link between these correlations and asthma pathogenesis remains to be investigated. 

Different mRNA expression profiles of T cell-related cytokines are observed in the mild and severe models for allergic asthma. To our knowledge, this is the first report in which *cytokine mRNA* expression is measured in mouse whole lung tissue. In severe asthma, allergen sensitization and challenge increased *Il6*, *Il5*, and *Il10* expression. These results are in line with findings in human asthmatics [11]. Of particular interest, *Il10* showed the largest change in mRNA expression in mild asthma. This profound 13-fold change in *Il10 *expression might be necessary to limit the inflammation in the airways and to counter the effects of the other cytokines on disease progression as previously described [38]. This hypothesis is supported by the findings in the severe model, in which *Ifn*γ** showed the largest change in expression suggesting a Th1-skewed response. 

Th2 cells play a key role in the pathogenesis of allergic asthma, and asthmatic patients have been reported to have Th1/Th2 imbalances as well as disturbed Th17 (Th17)/Treg balances. In mild asthma, expression of *Foxp3* was the most prominent. In severe asthma, *Foxp3* and *Tbet* showed the highest expression in lungs of allergen-sensitized and challenged mice. These findings are in line with our cytokine expression data (described above) and are supported by the proposed role of Tregs and *Il10 *in the airways [38]. Intriguingly, allergen sensitization and challenge resulted in a strong Treg response in both models. Accordingly, *Foxp3* showed the largest change in expression in the mild model. In severe asthma, the largest change in expression was found in the Th1-related transcription factor, *Tbet*, and this result is in contrast with data obtained from asthmatic patients [30, 32, 33], possibly due to measurement of mRNA expression in mouse whole lung tissue instead of in PBMCs of asthmatics and/or measurement of mRNA expression instead of protein expression. It has been reported that both *in vivo* and *in vitro Gata3 *can inhibit *Foxp3* gene induction by directly binding to the *Foxp3* promoter in mice [39]. An opposite action of *Foxp3* might also be possible, and our results could be explained by a counter-regulatory mechanism of Treg/Th1 to suppress the Th2 immune response. Interestingly, the mRNA expression of *Tbet* was strongly correlated with the expression of innate cytokines (*Il2*, *Il6,* and *Tnf*α**) and Th2 cytokines (*Il4*, *Il5, *and *Il13*), but not with Th1 cytokine (*Ifn*γ**). In severe asthma, *Ror*γ*t* expression was significantly correlated with *Il6* expression, but not in mild asthma. Expression of *Foxp3* was significantly correlated with *Il10 *expression in both models. *IL2*, *IL4*, *IL5*, *IL6*, *IL13,* and *TNF*α**have been reported to enhance asthma in humans, and Th1 cells have been shown to suppress Th2 cells through the release of *IFN-*γ*. *Additionally, Tregs suppress other Th cell effector functions through the release of *IL-10 *[11]. No correlations were found between *Gata3* and Th2 cytokines expression suggesting a counter-regulatory mechanism of Treg/Th1 to suppress the Th2 immune response as described previously.

To our knowledge, our results demonstrated for the first time that in mild and severe models for experimental asthma, immune and inflammatory responses are regulated differently. We showed that in mild and severe allergic asthma different mRNA expression of TLRs, NLRs and T cell-specific cytokines and transcription factors is observed. How this determines asthma severity remains to be investigated. This study adds to our understanding of the allergic characteristics of mild and severe allergic asthma which can contribute to the identification of phenotype-specific therapeutic targets. 

## Supplementary Material

Toll-like receptor (TLR), Nod-like receptor (NLR), ribosomal protein S13 (RPS13, reference gene) and T-cell transcription factor forward and reverse primers used for quantitative real-time PCR analysis.

## Figures and Tables

**Table 1:** Correlations between TLR and NLR mRNA expression in whole lung tissue and the total cell number in BAL fluid during mild and severe allergic asthma models.

TLR/NLR	Model	Correlation
*Tlr1 *	Mild	*P* > 0.05
*Tlr1 *	Severe	*r* ^2^ = 0.74
*Tlr3 *	Mild	*P* > 0.05
*Tlr3 *	Severe	*r* ^2^ = 0.61
*Tlr6 *	Mild	*P* > 0.05
*Tlr6 *	Severe	*r* ^2^ = 0.62
*Tlr9 *	Mild	*P* > 0.05
*Tlr9 *	Severe	*r* ^2^ = 0.36
*Tlr11 *	Mild	*P* > 0.05
*Tlr11 *	Severe	*r* ^2^ = 0.95
*Tlr13 *	Mild	*P* > 0.05
*Tlr13 *	Severe	*r* ^2^ = 0.68
*Nod1 *	Mild	*P* > 0.05
*Nod1 *	Severe	*r* ^2^ = 0.43
*Nod2 *	Mild	*P* > 0.05
*Nod2 *	Severe	*r* ^2^ = 0.39

Correlation is calculated using linear regression analysis. *R* square values are shown for the statistically significant correlations; *P* > 0.05 represents nonsignificant correlations.

**Table 2:** Relative T-cell *cytokine mRNA* expression in mouse whole lung tissue during mild and severe allergic asthma.

Cytokine	Model	Control a	Sensitized only b	Challenged only c	Sensitized and challenged
*Il2 *	Mild	1.00 ± 0.02	2.24 ± 1.41	12.96 ± 9.17	11.63 ± 11.32
*Il2 *	Severe	1.00 ± 0.14	1.21 ± 0.20	1.53 ± 0.55	90.66 ± 37.70
*Il4 *	Mild	1.00 ± 0.21	1.95 ± 0.49	12.29 ± 8.58	27.71 ± 27.25
*Il4 *	Severe	1.00 ± 0.05	1.00 ± 0.11	0.82 ± 0.31	43.38 ± 38.41
*Il5 *	Mild	1.00 ± 0.01	2.91 ± 2.08	8.09 ± 4.63	30.60 ± 0.00^d^
*Il5 *	Severe	1.00 ± 0.22	0.62 ± 0.11	0.56 ± 0.02	8.02 ± 0.89^a,b,c^
*Il6 *	Mild	1.00 ± 0.34	0.48 ± 0.09	2.94 ± 1.01	5.80 ± 3.49
*Il6 *	Severe	1.00 ± 0.25	0.90 ± 0.46	1.45 ± 0.83	33.44 ± 4.31^a,b,c^
*Il10 *	Mild	1.00 ± 0.28	2.22 ± 0.37	1.55 ± 0.49	18.93 ± 0.67^a,b,c^
*Il10 *	Severe	1.00 ± 0.33	1.24 ± 0.14	0.89 ± 0.43	25.94 ± 8.76^a,b,c^
*Il13 *	Mild	1.00 ± 0.32	2.75 ± 2.63	11.44 ± 7.40	9.58 ± 2.20
*Il13 *	Severe	1.00 ± 0.28	0.98 ± 0.21	0.87 ± 0.37	64.21 ± 34.25
*Tnf*α**	Mild	1.00 ± 0.11	1.01 ± 0.11	2.39 ± 0.87	4.83 ± 4.02
*Tnf*α**	Severe	1.00 ± 0.29	0.86 ± 0.14	1.17 ± 0.25	8.78 ± 4.87
*Ifn*γ**	Mild	1.00 ± 0.31	3.51 ± 2.77	25.26 ± 21.10	8.78 ± 1.16
*Ifn*γ**	Severe	1.00 ± 0.00	0.75 ± 0.14	0.74 ± 0.26	82.32 ± 60.37

The values shown in the table are relative to the *cytokine mRNA* expression levels in control mice. Data is shown as mean ± SEM. Statistical significance of differences was tested using Bonferroni's post hoc test after one-way ANOVA. ^a^ *P* < 0.05 compared to control mice; ^b^ *P* < 0.05 compared to sensitized only mice; ^c^ *P* < 0.05 compared to challenged only mice; ^d^a single value.

**Table 3:** Relative T-cell transcription factor mRNA expression in mouse whole lung tissue during mild and severe allergic asthma.

Transcription factor	Model	Control a	Sensitized only b	Challenged only c	Sensitized and challenged
*Tbet *	Mild	1.00 ± 0.40	1.06 ± 0.35	4.56 ± 2.23	2.32 ± 0.51
	Severe	1.00 ± 0.19	1.05 ± 0.31	1.45 ± 0.02	15.94 ± 5.09^a,b,c^
*Gata3 *	Mild	1.00 ± 0.01	0.68 ± 0.16	1.10 ± 0.14	1.44 ± 0.45
	Severe	1.00 ± 0.17	0.67 ± 0.08	0.57 ± 0.06	0.84 ± 0.21
*Ror*γ*t *	Mild	1.00 ± 0.11	0.67 ± 0.33	0.72 ± 0.15	0.35 ± 0.02
	Severe	1.00 ± 0.02	0.71 ± 0.19	1.02 ± 0.14	0.117^d^
*Foxp3 *	Mild	1.00 ± 0.31	1.87 ± 0.50	0.95 ± 0.34	8.55 ± 2.82^a^
	Severe	1.00 ± 0.11	0.92 ± 0.21	2.03 ± 0.39	17.11 ± 1.06^a,b,c^

Ratio					

*Gata3/Tbet *	Mild	1.00 ± 0.10	0.64 ± 0.26	0.24 ± 0.03	0.10 ± 0.03^a,b^
	Severe	1.00 ± 0.17	0.63 ± 0.07	0.30 ± 0.03	0.05 ± 0.01^a,b^
*Foxp3/Ror*γ*t *	Mild	1.00 ± 0.31	2.78 ± 0.63	4.33 ± 3.05	37.12 ± 12.24^a,b,c^
	Severe	1.00 ± 0.11	1.29 ± 0.29	2.00 ± 0.38	439.08 ± 27.31^a,b,c^
*Foxp3/Gata3 *	Mild	1.00 ± 0.31	2.77 ± 0.63	2.83 ± 1.98	5.95 ± 1.96^a^
	Severe	1.00 ± 0.11	1.37 ± 0.31	3.55 ± 0.67	20.40 ± 1.27^a,b,c^
*Foxp3/Tbet *	Mild	1.00 ± 0.31	1.77 ± 0.50	0.69 ± 0.48	0.61 ± 0.20
	Severe	1.00 ± 0.11	0.87 ± 0.20	1.07 ± 0.20	1.07 ± 0.07

*Tbet, Gata3, Ror*γ*t,* and *Foxp3* represent Th1, Th2, Th17, and Treg cells, respectively. The values shown in the table are relative to transcription factor mRNA expression levels in control mice. Data is shown as mean ± SEM. Ratios for Th2/Th1 (*Gata3/Tbet*), Treg/Th17 (*Foxp3/Ror*γ*t*), Treg/Th2 (*Foxp3/Gata3*), and Treg/Th1 (*Foxp3/Tbet*) mRNA expression are also shown. The mean ratio was calculated by dividing the individual expression values for the first transcription factor (numerator) by the mean expression value for the second transcription factor (denominator). Statistical significance of differences was tested using Bonferroni's post hoc test after one-way ANOVA. ^a^ *P* < 0.05 compared to control mice; ^b^ *P* < 0.05 compared to sensitized only mice; ^c^ *P* < 0.05 compared to challenged only mice; ^d^a single value.

**Table 4:** Correlation between T-cell transcription factor mRNA expression and T-cell *cytokine mRNA* expression in whole lung tissue during mild and severe allergic asthma.

Transcription factor	*Il2 *	*Il4 *	*Il5 *	*Il6 *	*Il10 *	*Il13 *	*Tnf*α**	*Ifn*γ**
*Tbet*/mild	*r* ^2^ = 0.60	*r* ^2^ = 0.91	*r* ^2^ = 0.93	*r* ^2^ = 0.82	*P* > 0.05	*r* ^2^ = 0.86	*r* ^2^ = 0.88	*P* > 0.05
*Tbet*/severe	*r* ^2^ = 0.92	*P* > 0.05	*P* > 0.05	*r* ^2^ = 0.97	*r* ^2^ = 0.96	*P* > 0.05	*P* > 0.05	*P* > 0.05
*Gata3*/mild	*P* > 0.05	*P* > 0.05	*P* > 0.05	*P* > 0.05	*P* > 0.05	*P* > 0.05	*P* > 0.05	*P* > 0.05
*Gata3*/severe	*P* > 0.05	*P* > 0.05	*P* > 0.05	*P* > 0.05	*P* > 0.05	*P* > 0.05	*P* > 0.05	*P* > 0.05
*Ror*γ*t*/mild	*P* > 0.05	*P* > 0.05	*P* > 0.05	*P* > 0.05	*r* ^2^ = 0.53	*P* > 0.05	*P* > 0.05	*P* > 0.05
*Ror*γ*t*/severe	*r* ^2^ = 0.50	*r* ^2^ = 0.51	*r* ^2^ = 0.42	*r* ^2^ = 0.46	*r* ^2^ = 0.54	*r* ^2^ = 0.44	*P* > 0.05	*P* > 0.05
*Foxp3*/mild	*r* ^2^ = 0.63	*r* ^2^ = 0.52	*r* ^2^ = 0.88	*r* ^2^ = 0.58	*r* ^2^ = 0.83	*P* > 0.05	*r* ^2^ = 0.46	*P* > 0.05
*Foxp3*/severe	*r* ^2^ = 0.98	*r* ^2^ = 0.92	*r* ^2^ = 0.98	*r* ^2^ = 0.99	*r* ^2^ = 0.58	*r* ^2^ = 0.98	*P* > 0.05	*r* ^2^ = 0.80

Correlation is calculated using linear regression analysis. *R* square values are shown for the statistically significant correlations; *P* > 0.05 represents nonsignificant correlations.
