# Supplementary material for: Differential Regulation of Inflammation and Immunity in Mild and Severe Experimental Asthma
Source: Mediators Inflamm. 2013 May 27;2013:808470. doi: 10.1155/2013/808470 (PMC3679512; doi:10.1155/2013/808470)
Supplement: Supplementary file 1 — Toll-like receptor (TLR), Nod-like receptor (NLR), ribosomal protein S13 (RPS13, reference gene) and T-cell transcription factor forward and reverse primers used for quantitative real-time PCR analysis. [file 808470.f1.doc]

***Supplementary Table 1:*** *Forward and reverse primers used for quantitative real-time PCR analysis*

| **Molecule** | **Forward primer** | **Reverse primer** |
| --- | --- | --- |
| ***TLR1*** | GGTGTTAGGAGATGCTTATGGGG | GATGTTAGACAGTTCCAAACCGA |
| ***TLR2*** | CCAGACACTGGGGGTAACATC | CGGATCGACTTTAGACTTTGGG |
| ***TLR3*** | GGGGTCCAACTGGAGAACCT | CCGGGGAGAACTCTTTAAGTGG |
| ***TLR4*** | GCCTTTCAGGGAATTAAGCTCC | AGATCAACCGATGGACGTGTAA |
| ***TLR5*** | TCAGACGGCAGGATAGCCTT | AATGGTCAAGTTAGCATACTGGG |
| ***TLR6*** | GACTCTCCCACAACAGGATACG | TCAGGTTGCCAAATTCCTTACAC |
| ***TLR7*** | TCTTACCCTTACCATCAACCACA | CCCCAGTAGAACAGGTACACA |
| ***TLR8*** | GGCACAACTCCCTTGTGATT | CATTTGGGTGCTGTTGTTTG |
| ***TLR9*** | ACTCCGACTTCGTCCACCT | GGCTCAATGGTCATGTGGCA |
| ***TLR11*** | AAAACCAGACAACATCACAA | GCATCCCAAATAGATAGAGG |
| ***TLR12*** | GAACTCTTGGATCCCCTAC | GGCAGAAGTTCCTCTATCAC |
| ***TLR13*** | ATCTCAGGAACAAAAGATGG | TGTTCCCATAGACATCAAAA |
| ***NOD1*** | GAAGGCACCCCATTGGGTT | AATCTCTGCATCTTCGGCTGA |
| ***NOD2*** | CCGCTTTCTACTTGGCTGTC | GTGATTTGCAGGTTGTGTGG |
| **T-bet** | GCCAGCCAAACAGAGAAGAC | AAATGTGCACCCTTCAAACC |
| **GATA-3** | GCGGTACCTGTCTTTTTCGT | CACACAGGGGCTAACAGTCA |
| **Foxp3** | CACTGGGCTTCTGGGTATGT | AGACAGGCCAGGGGATAGTT |
| **RORγt** | TGCAAGACTCATCGACAAGG | AGGGGATTCAACATCAGTGC |
| **RPS13** | GTCCGAAAGCACCTTGAGAG | AGCAGAGGCTGTGGATGACT |
